# Combined effect of menopause and cardiovascular risk factors on death and cardiovascular disease: a cohort study

**DOI:** 10.1186/s12872-021-01919-5

**Published:** 2021-02-23

**Authors:** Yan Li, Dong Zhao, Miao Wang, Jia-yi Sun, Jun Liu, Yue Qi, Yong-chen Hao, Qiu-ju Deng, Jue Liu, Jing Liu, Min Liu

**Affiliations:** 1 Department of Epidemiology and Biostatistics, School of Public Health, Peking University, 38 Xue Yuan Road, Haidian District, Beijing, 100191 China; 2 Department of Epidemiology, Beijing An Zhen Hospital, Capital Medical University, Beijing Institute of Heart, Lung and Blood Vessel Diseases, Beijing, China

**Keywords:** Menopause, Risk factor, Cardiovascular disease, Stroke

## Abstract

**Background:**

Observational studies suggest that early menopause is associated with increased risk of death and cardiovascular disease (CVD); however, the results of these studies have been inconsistently. We aimed to assess the association of menopause with death and CVD and whether this association was modified by cardiovascular risk factors.

**Methods:**

The study population was women age 35–64 years living in two communities of Beijing who were enrolled in the Chinese Multi-provincial Cohort Study in 1992. Participants were followed until first cardiovascular event, death, or the end of follow-up (2018). Self-reported age at menopause was recorded. Multivariate Cox regression models were used to estimate the hazard ratios (HRs) and 95% confidence intervals (CIs) of death and CVD after adjusting for baseline covariates of age, family history of CVD, and white blood cell count, as well as time-varying covariates of menopause, use of oral estrogen, and conventional risk factors. Additionally, we assessed the combined effect of age at menopause and risk factors on the primary endpoint.

**Results:**

Of 2104 eligible women, 124 died and 196 had a first CVD event (33 fatal CVD and 163 non-fatal CVD). Compared with women who experienced menopause at age 50–51 years, the risk of death was higher in women with menopause at age 45–49 years (HR 1.99, 95% CI 1.24–3.21; *P* = 0.005), and the risk of ischemic stroke was higher in women with menopause at age < 45 years (HR 2.16, 95% CI 1.04–4.51; *P* = 0.04) and at age 45–49 years (HR 2.05, 95% CI 1.15–3.63; *P* = 0.01). Women who had menopause before age 50 years and at least one elevated risk factor at baseline had a higher risk of death (HR 11.10, 95% CI 1.51–81.41; *P* = 0.02), CVD (HR 3.98, 95% CI 1.58–10.01; *P* = 0.003), ischemic CVD (HR 4.53, 95% CI 1.63–12.62; *P* = 0.004), coronary heart disease (HR 8.63, 95% CI 1.15–64.50; *P* = 0.04), and stroke (HR 2.92, 95% CI 1.03–8.29; *P* = 0.04) than those with menopause at age 50–51 years and optimal levels of all risk factors.

**Conclusions:**

Earlier menopause may predict death and ischemic stroke. Furthermore, there is a combined effect of earlier menopause and elevated risk factors on death and CVD.

## Introduction

Menopause is a fundamental biological event in a woman’s life and is the final step in the process referred to as ovarian aging. Most women experience menopause between age 40 and 60 years; the average age at menopause is 51 years [1]. The variation in age at menopause is very similar across different populations [2]. There are up to 10% of women experiencing menopause before age 45 years [3]. Menopause is considered as a marker not only for reproductive aging but also for general health and somatic aging in women [1]. Cardiovascular disease (CVD) is the leading cause of death among women worldwide. A report from the Atherosclerosis Risk in Communities (ARIC) study showed that CVD mortality rates are increasing among women younger than 55 years of age [4]. Early menopause has been reported to be associated with increased risk of future CVD events among women in Western countries [5, 6], which suggests the importance of identifying a potential target population for early cardiovascular risk stratification.

Results from one meta-analysis indicated that menopause before age 50 years was associated with a 25% increased risk of CVD [7]. Another meta-analysis indicated that women with menopause before age 45 years had a 50% increased risk of coronary heart disease (CHD), but the association between age at menopause and incident stroke was inconsistent [6]. Women generally spend the last third of their life in the postmenopausal stage [8]; therefore, women with earlier menopause are more likely to suffer effects on their vascular health. However, limited robust data are available on the long-term risk of death and developing CVD among women who experience early menopause, and most evidence is derived from Western populations. It is therefore necessary to confirm the association of early menopause with death and CVD in other ethnic populations. Additionally, menopause is also usually associated with other conditions, including obesity [9], hypertension [10], diabetes [11], and dyslipidemia [12]; moreover, how these cardiovascular risk factors affect the association of menopause with CVD and death need to be confirmed.

The objective of this study was to prospectively examine the association of early menopause with the risk of death and incidence of CVD in women from two communities in Beijing. The combined effect of early menopause and conventional cardiovascular risk factors was also explored. We aimed to provide evidence for the association of female reproductive aging with cardiovascular system health and mortality.

## Materials and methods

### Study population

Participants were residents of two communities in Beijing and were part of the Chinese Multi-provincial Cohort Study (CMCS). The CMCS is a multicenter, prospective, population-based cohort study on the determinants of CVD. Details of the study have been published previously [13]. The baseline survey began in 1992. A standardized questionnaire modified according to the WHO-MONICA protocol for risk factor survey was used to collect information on demographic characteristics, smoking habit, personal medical history, and medical therapy [14]. Participants signed the written informed consents, completed questionnaires, and underwent physical measurements as well as phlebotomy. The ethics committee of Beijing An Zhen Hospital approved the study.

**Fig. 1:**
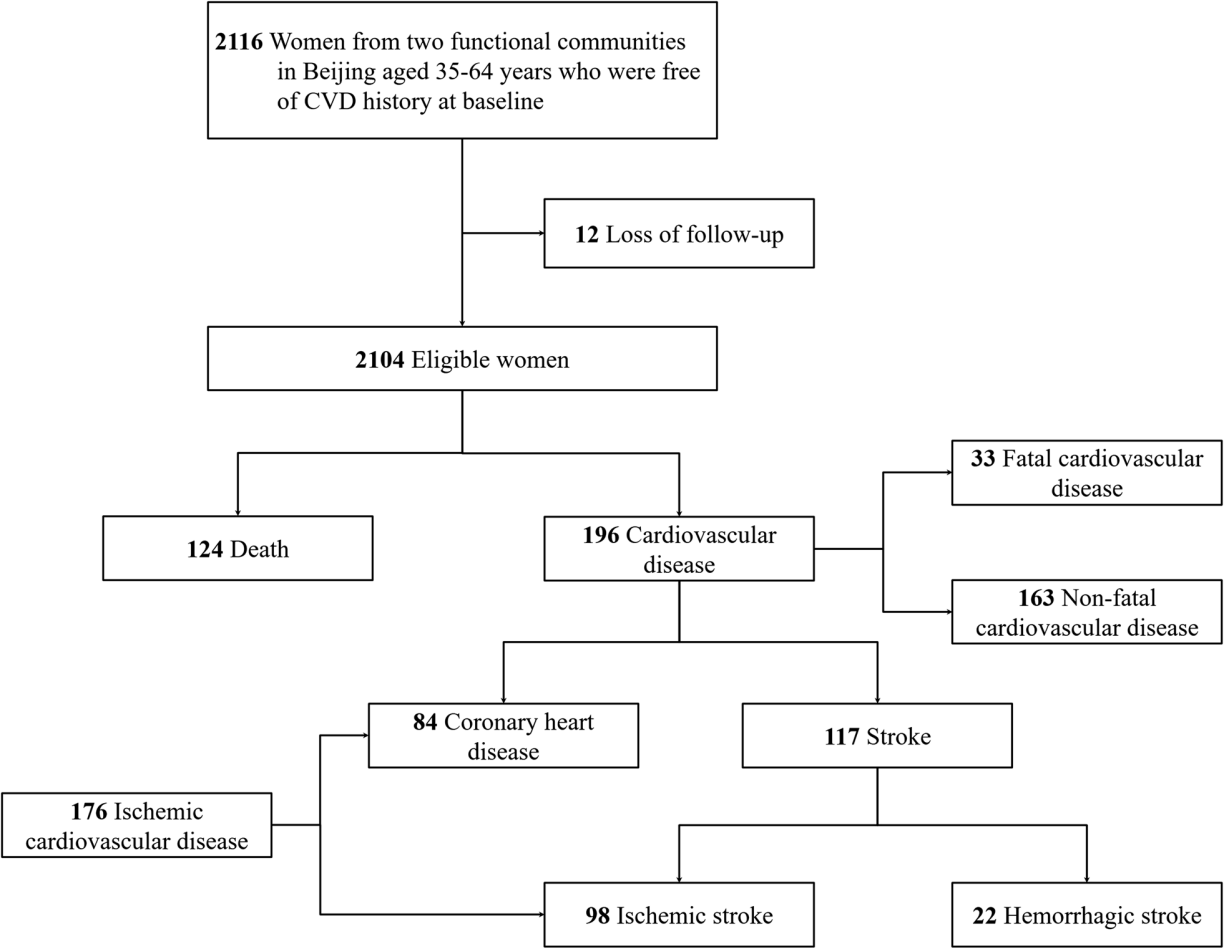
Study profile. *CVD* cardiovascular disease

A total of 2116 women aged 35–64 years who were free of CVD history at baseline were included in the present study. All participants had a follow-up visit every 1–2 years from the baseline examination through the first CVD event, death, or the end of follow-up (2018). After excluding women who were lost to follow-up, 2104 women were eligible for inclusion. Figure 1 shows the study profile.

### Exposure

Women reported their natural menopause status (premenopausal or postmenopausal), and if postmenopausal, their age at natural menopause. All women were in postmenopausal status until the end of follow-up. Age at menopause was categorized as less than 45 years (early menopause), 45–49 years (relatively early menopause), 50–51 years (reference), 51 years or older (relatively late menopause). When conducting analyses of the combined effect of age at menopause and cardiovascular risk factors at baseline, age at menopause was also categorized into three different menopausal age groups (< 50 years, 50–51 years, and > 51 years).

Using an existing menopause staging system [15], baseline menopausal status was categorized as reproductive (> 3 years before menopause), menopausal transition (≤ 3 years before menopause and ≤ 0 years since menopause)/perimenopause (≤ 3 years before menopause and ≤ 1 years since menopause), early postmenopause (> 0/1 years and ≤ 6 years since menopause), and late postmenopause (> 6 years since menopause).

### Outcome

The primary study endpoint was death or the occurrence of a first CVD event (including fatal CVD or non-fatal CVD), defined as a composite outcome of incident CHD or stroke (including ischemic stroke or hemorrhagic stroke). Additionally, a composite endpoint of incident CHD or ischemic stroke was also defined as ischemic CVD in the analysis. Acute CVD events were ascertained according to the diagnostic criteria of the World Health Organization-MONICA protocol [16]. Coronary events were diagnosed based on symptoms, electrocardiography recordings, serum myocardial enzymes, autopsy findings, and history of CHD. Stroke events were diagnosed as rapidly developing signs of focal (or global) disturbances in cerebral function lasting more than 24 h (unless interrupted by surgery or death) with no apparent non-vascular cause [17]. All suspected CVD events were reviewed by a team of physicians using data from the original medical records. All causes of death were registered based on the death certificate issued by the physician and were double-checked by researchers using the original medical or death records [18].

### Covariates

The following factors were included in the analyses as covariates: age at baseline, family history of CVD, white blood cell count, time-varying menopause, use of oral estrogen due to menopause, smoking status (regular smoker, occasional smoker, never smoker, or quit smoking for > 1 year), body mass index (BMI), systolic blood pressure (SBP), diastolic blood pressure (DBP), fasting blood glucose (FG), total cholesterol (TC), triglycerides (TG), low-density lipoprotein cholesterol (LDL-C), and high-density lipoprotein cholesterol (HDL-C). Optimal risk factor levels were defined as never smoker or quit smoking for > 1 year, BMI ≤ 24 kg/m^2^, SBP < 130 mmHg, DBP < 80 mmHg, FG < 6.1 mmol/L, LDL-C < 3.37 mmol/L, TC < 5.17 mmol/L, TG < 1.70 mmol/L, and HDL-C > 1.04 mmol/L. Non-optimal risk factor levels were defined as occasional smoker, BMI 24–28 kg/m^2^, SBP 130–140 mmHg, DBP 80–90 mmHg, FG 6.1–7.0 mmol/L, LDL-C 3.37–4.14 mmol/L, TC 5.17–6.20 mmol/L, TG 1.70–2.26 mmol/L, and HDL-C 0.91–1.04 mmol/L. Elevated risk factor levels were defined as regular smoker, BMI > 28 kg/m^2^, SBP ≥ 140 mmHg, DBP ≥ 90 mmHg, FG ≥ 7.0 mmol/L, LDL-C ≥ 4.14 mmol/L, TC ≥ 6.20 mmol/L, TG ≥ 2.26 mmol/L, and HDL-C ≤ 0.91 mmol/L.

### Statistical analysis

Baseline continuous variables were compared using one-way analysis of variance and categorical variables were compared using the χ^2^ test. Cumulative incidence of death and CVD events were estimated using the Kaplan–Meier method. Multivariate Cox regression models were used to estimate hazard ratios (HRs) and 95% confidence intervals (CIs) for death and CVD events associated with menopause. For women who died or had first occurrence of a CVD event, follow-up time was calculated as their age at death or first event minus their entry age; for women without death and CVD endpoint events, follow-up time was identified as their age at last follow-up minus their entry age. Menopause at age 50–51 years was used as the reference category. In the fully adjusted models, HRs and 95% CIs were adjusted for: time-varying covariates including menopause, use of oral estrogen owing to menopause, smoking, BMI, SBP, DBP, FG, TC, TG, LDL-C, and HDL-C; as well as baseline covariates including age, family history of CVD, and white blood cell count. Sensitivity analysis was conducted after excluding cases of premature menopause (age < 40 years) to test the robustness of the findings.

To examine whether earlier menopause was associated with greater risk of death or first occurrence of a CVD event when combined with elevated risk factors compared with later menopause combined with optimal risk factor levels, we stratified the analyses using a conventional risk factor grade at baseline based on menopausal age groups: menopause at age < 50, 50–51, and > 51 years combined with risk factor grading at all risk factor levels (optimal, ≥ 1 non-optimal risk factor, and ≥ 1 elevated risk factor). Women with menopause at age 50–51 years and all risk factors at optimal levels were regarded as the reference group. In the combined effects models, HRs and 95% CIs were adjusted for: time-varying menopause and use of oral estrogen owing to menopause, as well as baseline age, family history of CVD, and white blood cell count. All statistical analyses were performed using IBM SPSS version 22.0 (IBM Corp., Armonk, NY, USA).

## Results

### Baseline characteristics of the study cohort: descriptive analysis

The final cohort consisted of 2104 women. The mean (standard deviation, SD) age at baseline was 45.2 (7.8) years. There were 1252 reproductive women whose mean age (SD) was 39.9 (3.5) years, 309 perimenopausal women with mean age (SD) 48.9 (4.4), 283 early postmenopausal women with mean age (SD) 53.0 (3.9) years, and 260 late postmenopausal women with mean age (SD) 57.6 (4.4) years at baseline (Table 1). All women reached postmenopausal status during the follow-up period. The mean (SD) age at menopause was 50.0 (3.8) years. A total of 790 (37.5%) women had menopause at age 45–49 years and 160 (7.6%) women had menopause at age < 45 years, among whom 40 (1.9%) experienced menopause at age < 40 years (premature menopause). Women were followed up for a mean 25.6 years (SD, 3.2) to death and 24.7 years (SD, 4.8) to CVD incidence. During the follow-up period, 124 of the 2104 women died, and 196 occurred a CVD event (33 fatal CVD and 163 non-fatal CVD), among women who had incident CVD, 84 had CHD and 117 had stroke (98 with ischemic stroke and 22 with hemorrhagic stroke; Fig. 1).

**Table 1:** Baseline characteristics of the study cohort

	All (n = 2104)	Menopause staging at baseline					Age at menopause					
		Reproductive (n = 1252)	Perimenopause (n = 309)	Early postmenopause (n = 283)	Late postmenopause (n = 260)	*P* value	< 45 years (n = 160)	45–49 years (n = 790)	50–51 years (n = 506)	52–53 years (n = 375)	≥ 54 years (n = 273)	*P* value
Age at baseline (years old), mean ± SD	45.2 ± 7.8	39.9 ± 3.5	48.9 ± 4.4	53.0 ± 3.9	57.6 ± 4.4	< 0.001	47.0 ± 8.1	43.6 ± 7.1	46.2 ± 8.1	45.2 ± 7.8	46.7 ± 7.9	< 0.001
Body mass index (kg/m^2^), mean ± SD	23.9 ± 3.4	23.9 ± 3.5	24.2 ± 3.3	23.8 ± 3.2	23.6 ± 3.1	0.29	24.4 ± 3.5	23.9 ± 3.5	23.5 ± 3.3	24.0 ± 3.2	24.0 ± 3.4	0.02
Smoking, n (%)	18 (0.9)	7 (0.6)	6 (1.9)	2 (0.7)	3 (1.2)	0.11	0 (0.0)	7 (0.9)	8 (1.6)	1 (0.3)	2 (0.7)	0.19
Systolic blood pressure (mmHg), mean ± SD	119.2 ± 18.1	114.5 ± 15.2	123.0 ± 17.9	125.2 ± 18.9	130.9 ± 22.1	< 0.001	121.9 ± 18.6	118.1 ± 17.7	119.3 ± 19.0	119.3 ± 17.8	120.3 ± 17.6	0.12
Diastolic blood pressure (mmHg), mean ± SD	75.6 ± 10.5	73.6 ± 9.7	77.9 ± 11.1	78.4 ± 10.8	79.9 ± 11.1	< 0.001	77.2 ± 10.7	75.1 ± 10.5	75.6 ± 10.7	75.5 ± 10.3	76.4 ± 10.5	0.11
Hypertension, n (%)	347 (16.5)	119 (9.5)	71 (23.1)	74 (26.1)	83 (31.9)	< 0.001	31 (19.4)	124 (15.7)	82 (16.2)	58 (15.5)	52 (19.0)	0.57
Total cholesterol (mmol/L), mean ± SD	4.67 ± 0.88	4.40 ± 0.77	4.84 ± 0.86	5.11 ± 0.82	5.29 ± 0.86	< 0.001	4.93 ± 0.83	4.61 ± 0.86	4.72 ± 0.89	4.66 ± 0.91	4.63 ± 0.86	0.001
Triglyceride (mmol/L), mean ± SD	1.09 ± 0.74	1.00 ± 0.64	1.14 ± 0.79	1.23 ± 0.98	1.36 ± 0.73	< 0.001	1.23 ± 0.74	1.00 ± 0.54	1.10 ± 0.87	1.15 ± 0.73	1.17 ± 0.93	< 0.001
High density lipoprotein cholesterol (mmol/L), mean ± SD	1.52 ± 0.36	1.51 ± 0.36	1.55 ± 0.36	1.58 ± 0.37	1.47 ± 0.34	0.001	1.52 ± 0.36	1.55 ± 0.36	1.51 ± 0.35	1.51 ± 0.35	1.47 ± 0.35	0.04
Low density lipoprotein cholesterol (mmol/L), mean ± SD	2.93 ± 0.83	2.69 ± 0.74	3.07 ± 0.82	3.29 ± 0.81	3.55 ± 0.82	< 0.001	3.16 ± 0.81	2.86 ± 0.81	2.99 ± 0.85	2.92 ± 0.88	2.92 ± 0.79	0.001
Fasting blood glucose (mmol/L), mean ± SD	5.28 ± 0.91	5.20 ± 0.89	5.33 ± 0.82	5.34 ± 0.78	5.58 ± 1.16	< 0.001	5.35 ± 0.96	5.28 ± 1.02	5.28 ± 0.76	5.28 ± 0.90	5.28 ± 0.84	0.94
Type 2 diabetes, n (%)	50 (2.4)	24 (1.9)	8 (2.6)	8 (2.8)	10 (3.9)	0.27	2 (1.3)	25 (3.2)	10 (2.0)	5 (1.3)	8 (2.9)	0.25

*SD* standard deviation

*P* values were calculated for continuous variables using analysis of variance or for categorical variables using the χ^2^ test

Compared with women who experienced menopause after age 50 years, those with menopause at age 45–49 years (relatively early menopause) were more likely to be younger and to have lower TC, TG, and LDL-C levels at the baseline; however, those who experienced menopause at age < 45 years (early menopause) were more likely to be older, overweight, and to have higher TC, TG, and LDL-C levels at the baseline (Table 1).

### Cumulative mortality and cumulative incidence rates of first CVD events

**Fig. 2:**
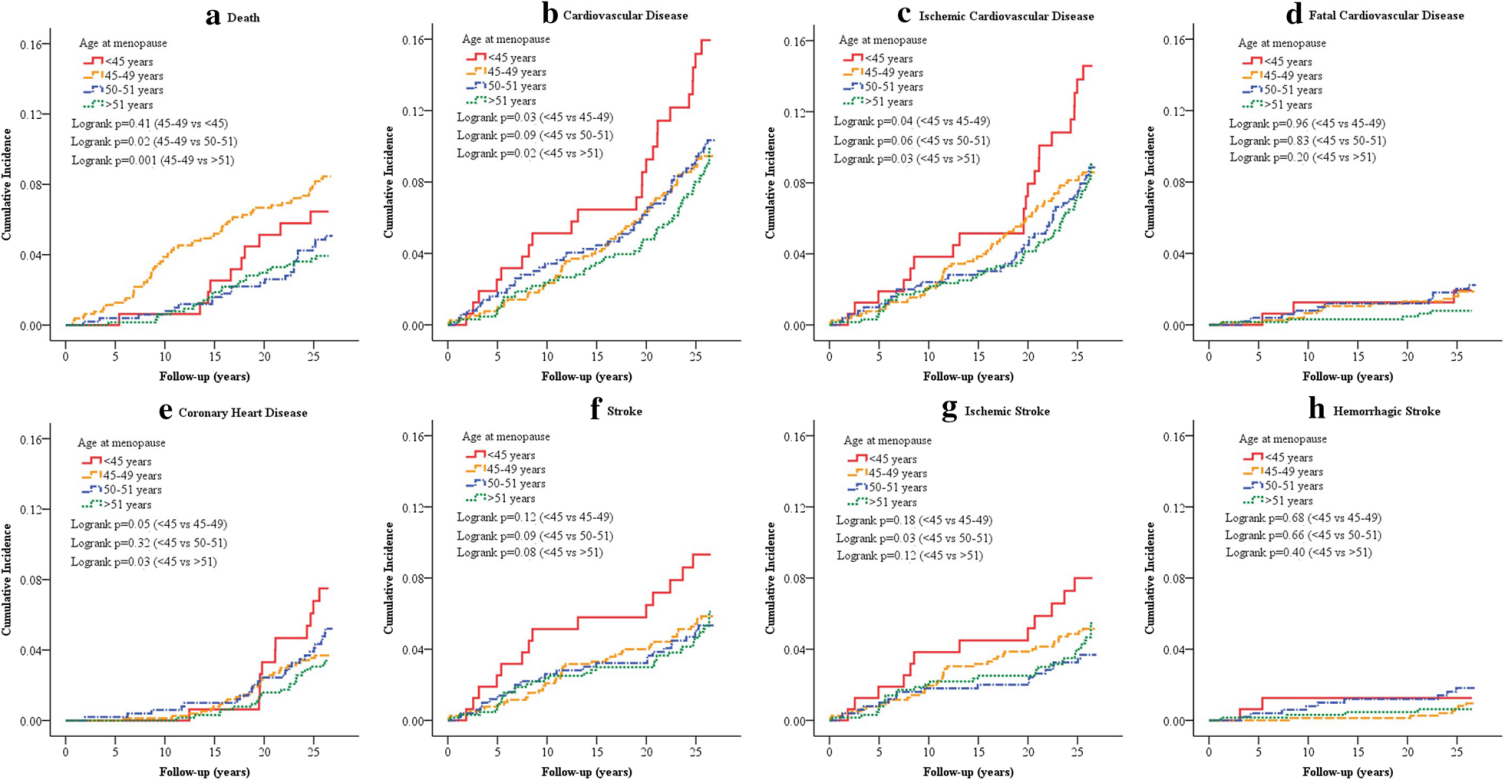
Cumulative incidence of death and cardiovascular events according to age at menopause

Women with menopause at age 45–49 years had a higher cumulative mortality rate than those with menopause at age 50–51 years (8.1% vs 4.9%, log rank *P* = 0.02) and those with menopause after age 51 years (8.1% vs 3.9%, log rank *P* = 0.001). The cumulative incidence rate of ischemic stroke was higher in women with menopause at age < 45 years than those with menopause at age 50–51 years (7.5% vs 3.6%, log rank *P* = 0.03). Additionally, the cumulative incidence rates of total CVD, ischemic CVD, and CHD were also higher among women who experienced menopause at age < 45 years than those with menopause at age 45–49 years and > 51 years (Fig. 2).

To examine whether years since menopause had an effect on the risks of death and first occurrence of a CVD event, we also calculated the cumulative mortality rate and cumulative incidence rate according to menopause staging in the baseline study. Compared with reproductive women at baseline, women at the late postmenopausal stage had a higher cumulative mortality rate (log rank *P* < 0.001) and higher cumulative incidence rates of cardiovascular events (total CVD, ischemic CVD, CHD, stroke, ischemic stroke: log rank *P* < 0.001; Additional file 1: Figure S1).

### Age at menopause and risk of death and incident cardiovascular events

The person-year mortality rate was 2.4 and 3.2 per 1000 woman-years among women with menopause at age < 45 years and at age 45–49 years, respectively. The person-year incidence rates for total CVD, fatal CVD, ischemic CVD, CHD, stroke, ischemic stroke, and hemorrhagic stroke were 5.9, 0.7, 5.4, 2.7, 3.6, 3.0, and 0.5 per 1000 woman-years, respectively, among women with menopause at age < 45 years (Table 2). Compared with women who experienced menopause at age 50–51 years, the risks of death (HR 1.85, 95% CI 1.16–2.94; *P* = 0.01) and ischemic stroke (HR 2.08, 95% CI 1.20–3.59; *P* = 0.01) were higher among women with menopause at age < 50 years in the fully adjusted models (Table 3). Moreover, the death risk was only significant among women with menopause at age 45–49 years (HR 1.99, 95% CI 1.24–3.21; *P* = 0.005), and ischemic stroke risk was found to be increased among women with menopause at age < 45 years (HR 2.16, 95% CI 1.04–4.51; *P* = 0.04) and 45–49 years (HR 2.05, 95% CI 1.15–3.63; *P* = 0.01; Table 2). The risk estimates remained similar when sensitivity analyses were conducted; that is, after excluding women who experienced menopause at age < 40 years, those with menopause at age 45–49 years had a 2.01 times higher risk of death (95% CI 1.25–3.23; *P* = 0.004) and 2.07 times higher risk of ischemic stroke (95% CI 1.17–3.68; *P* = 0.01) than women with menopause at age 50–51 years (Additional file 1: Table 1).

**Table 2:** Hazard ratios and 95%CIs for death and cardiovascular events associated with age at menopause (four groups) in women

Age at menopause	Women	Events	Woman-years	Events per 1000 person-years	Adjusted HR (95% CI)	*P* value^a^
Death						
< 45 years old	160	10	4107	2.4	1.32 (0.63, 2.76)	0.46
45–49 years old	790	64	19,880	3.2	1.99 (1.24, 3.21)	0.005
50–51 years old	506	25	13,088	1.9	1.00 (reference)	0.001
> 51 years old	648	25	16,767	1.5	0.79 (0.45, 1.38)	0.40
Cardiovascular disease						
< 45 years old	160	23	3893	5.9	1.46 (0.89, 2.41)	0.14
45–49 years old	790	68	19,200	3.5	1.18 (0.81, 1.72)	0.39
50–51 years old	506	49	12,611	3.9	1.00 (reference)	0.22
> 51 years old	648	56	16,269	3.4	0.91 (0.62, 1.34)	0.63
Fatal cardiovascular disease						
< 45 years old	160	3	4101	0.7	0.86 (0.24, 3.09)	0.82
45–49 years old	790	14	19,802	0.7	1.19 (0.53, 2.69)	0.67
50–51 years old	506	11	13,038	0.8	1.00 (reference)	0.18
> 51 years old	648	5	16,747	0.3	0.38 (0.13, 1.09)	0.07
Ischemic cardiovascular disease						
< 45 years old	160	21	3917	5.4	1.56 (0.92, 2.65)	0.10
45–49 years old	790	62	19,230	3.2	1.28 (0.86, 1.91)	0.23
50–51 years old	506	42	12,697	3.3	1.00 (reference)	0.19
> 51 years old	648	51	16,316	3.1	0.97 (0.65, 1.47)	0.90
Coronary heart disease						
< 45 years old	160	11	4053	2.7	1.32 (0.65, 2.70)	0.44
45–49 years old	790	27	19,692	1.4	0.82 (0.47, 1.44)	0.50
50–51 years old	506	25	12,927	1.9	1.00 (reference)	0.25
> 51 years old	648	21	16,652	1.3	0.65 (0.36, 1.17)	0.15
Stroke						
< 45 years old	160	14	3939	3.6	1.72 (0.90, 3.31)	0.10
45–49 years old	790	43	19,402	2.2	1.50 (0.91, 2.46)	0.11
50–51 years old	506	26	12,804	2.0	1.00 (reference)	0.19
> 51 years old	648	34	16,397	2.1	1.06 (0.64, 1.78)	0.82
Ischemic stroke						
< 45 years old	160	12	3962	3.0	2.16 (1.04, 4.51)	0.04
45–49 years old	790	38	19,411	2.0	2.05 (1.15, 3.63)	0.01
50–51 years old	506	18	12,857	1.4	1.00 (reference)	0.05
> 51 years old	648	30	16,431	1.8	1.37 (0.76, 2.47)	0.29
Hemorrhagic stroke						
< 45 years old	160	2	4084	0.5	0.71 (0.15, 3.35)	0.67
45–49 years old	790	7	19,869	0.4	0.66 (0.24, 1.82)	0.42
50–51 years old	506	9	13,032	0.7	1.00 (reference)	0.37
> 51 years old	648	4	16,733	0.2	0.34 (0.10, 1.12)	0.08

Model adjusted for: time-varying covariates including menopause, use of oral estrogen owing to menopause, body mass index, smoking, systolic blood pressure, diastolic blood pressure, fasting glucose, total cholesterol, low density lipoprotein cholesterol, high density lipoprotein cholesterol, and triglyceride; baseline covariates including age, family history of cardiovascular disease, and white blood cell count

*HR* hazard ratio, *CI* confidence interval

^a^*P* values derived from Cox regression model for non-proportional hazards

**Table 3:** Hazard ratios and 95%CIs for death and cardiovascular events associated with age at menopause (three groups) in women

Age at menopause	Women	Events	Woman-years	Events per 1000 person-years	Adjusted HR (95% CI)	*P* value^a^
Death						
< 50 years old	950	74	23,987	3.1	1.85 (1.16, 2.94)	0.01
50–51 years old	506	25	13,088	1.9	1.00 (reference)	< 0.001
> 51 years old	648	25	16,767	1.5	0.79 (0.45, 1.37)	0.40
Cardiovascular disease						
< 50 years old	950	91	23,093	3.9	1.24 (0.87, 1.77)	0.23
50–51 years old	506	49	12,611	3.9	1.00 (reference)	0.17
> 51 years old	648	56	16,270	3.4	0.91 (0.62, 1.34)	0.63
Fatal cardiovascular disease						
< 50 years old	950	17	23,903	0.7	1.11 (0.51, 2.41)	0.79
50–51 years old	506	11	13,038	0.8	1.00 (reference)	0.10
> 51 years old	648	5	16,747	0.3	0.38 (0.13, 1.08)	0.07
Ischemic cardiovascular disease						
< 50 years old	950	83	23,147	3.6	1.35 (0.92, 1.96)	0.12
50–51 years old	506	42	12,697	3.3	1.00 (reference)	0.13
> 51 years old	648	51	16,316	3.1	0.97 (0.65, 1.47)	0.90
Coronary heart disease						
< 50 years old	950	38	23,744	1.6	0.93 (0.56, 1.56)	0.79
50–51 years old	506	25	12,927	1.9	1.00 (reference)	0.31
> 51 years old	648	21	16,652	1.3	0.65 (0.36, 1.17)	0.15
Stroke						
< 50 years old	950	57	23,341	2.4	1.55 (0.97, 2.48)	0.07
50–51 years old	506	26	12,804	2.0	1.00 (reference)	0.10
> 51 years old	648	34	16,397	2.1	1.06 (0.64, 1.78)	0.81
Ischemic stroke						
< 50 years old	950	50	23,373	2.1	2.08 (1.20, 3.59)	0.01
50–51 years old	506	18	12,857	1.4	1.00 (reference)	0.02
> 51 years old	648	30	16,431	1.8	1.37 (0.76, 2.47)	0.29
Hemorrhagic stroke						
< 50 years old	950	9	23,953	0.4	0.67 (0.26, 1.72)	0.40
50–51 years old	506	9	13,032	0.7	1.00 (reference)	0.21
> 51 years old	648	4	16,733	0.2	0.34 (0.10, 1.12)	0.08

Model adjusted for: time-varying covariates including menopause, use of oral estrogen owing to menopause, body mass index, smoking, systolic blood pressure, diastolic blood pressure, fasting glucose, total cholesterol, low density lipoprotein cholesterol, high density lipoprotein cholesterol, and triglyceride; baseline covariates including age, family history of cardiovascular disease, and white blood cell count

*HR* hazard ratio, *CI* confidence interval

^a^*P* values derived from Cox regression model for non-proportional hazards

Women with early postmenopause had a higher risk of CHD than those who were reproductive at baseline in the fully adjusted models (HR 2.87, 95% CI 1.24–6.65, *P* = 0.01, Additional file 1: Table 2; and HR 2.83, 95% CI 1.18–6.76, *P* = 0.02, Additional file 1: Table 3).

### Combined effect of age at menopause and conventional cardiovascular risk factors at baseline on risk of death and incident cardiovascular events

**Fig. 3:**
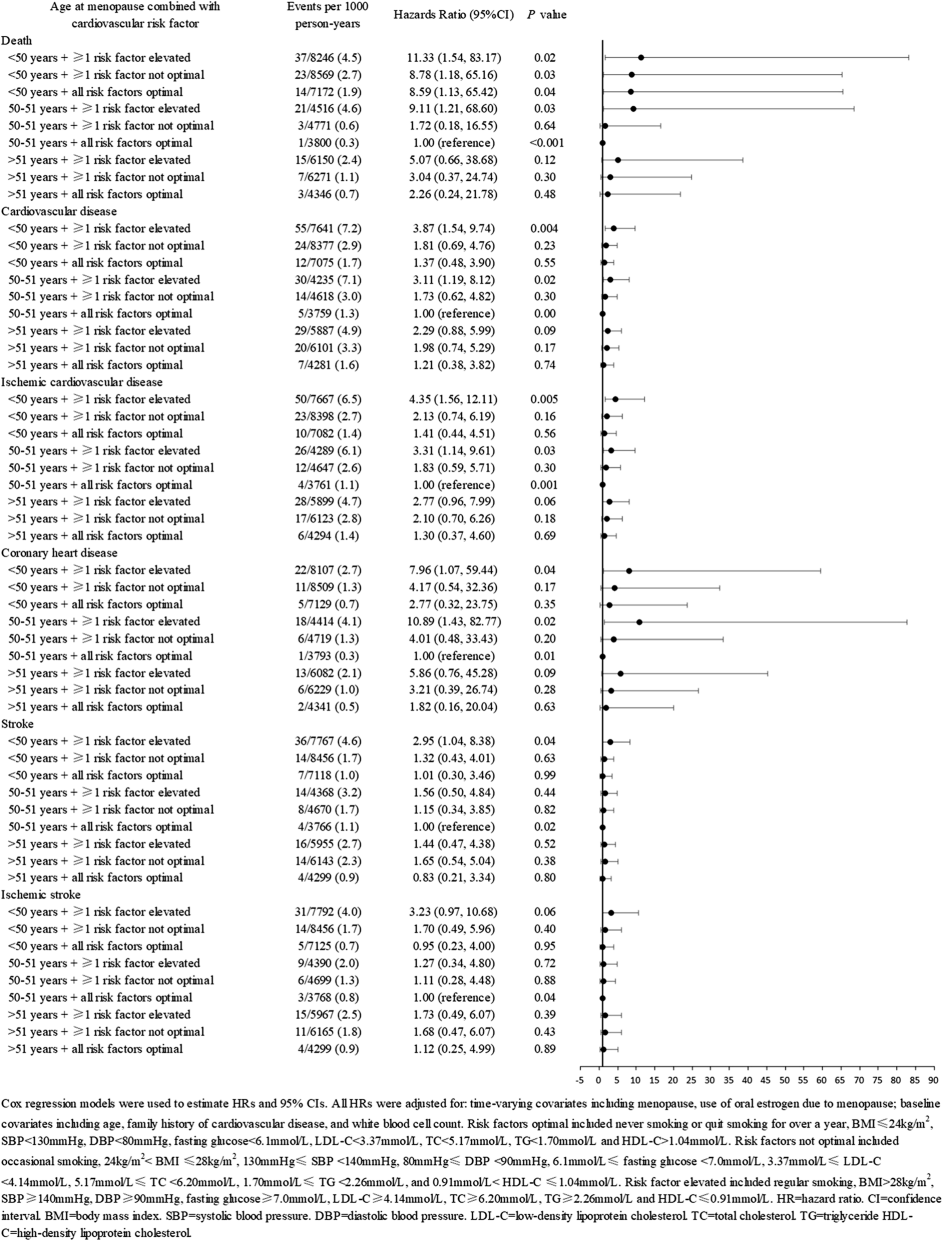
Combined effect of age at menopause and conventional cardiovascular risk factors at baseline on risk of death and cardiovascular events

The person-year mortality rate (4.5/1000 woman-years) and person-year incidence rates for total CVD (7.2/1000 woman-years), ischemic CVD (6.5/1000 woman-years), CHD (2.7/1000 woman-years), stroke (4.6/1000 woman-years), and ischemic stroke (4.0/1000 woman-years) among women with menopause at age < 50 years and at least one elevated cardiovascular risk factor were higher than the rates among those with menopause at age 50–51 years and all risk factors at optimal levels (Fig. 3).

A combined analysis of age at menopause and conventional cardiovascular risk factor grading status at baseline showed that compared with women who experienced menopause at age 50–51 years and had optimal levels of all risk factors, those who experienced menopause at age < 50 years and had at least one elevated cardiovascular risk factor at baseline had significantly increased risks of death (HR 11.33, 95% CI 1.54–83.17; *P* = 0.02), total CVD (HR 3.87, 95% CI 1.54–9.74; *P* = 0.004), ischemic CVD (HR 4.35, 95% CI 1.56–12.11; *P* = 0.005), CHD (HR 7.96, 95% CI 1.07–59.44; *P* = 0.04), and stroke (HR 2.95, 95% CI 1.04–8.38; *P* = 0.04), after adjustment for age, family history of CVD, white blood cell count at baseline, as well as time-varying menopause status and use of oral estrogen (Fig. 3).

## Discussion

Retrospective analysis of prospectively collected data from a cohort study indicated that the risk of death was nearly 2.0 times higher among women who had menopause at age 45–49 years and the risk of ischemic stroke was more than 2.1 times higher among women who had menopause at age < 45 years and more than 2.0 times higher among women with menopause at age 45–49 years than those who experienced menopause at age 50–51 years in the fully adjusted Cox models. Additionally, women who had menopause before age 50 years and at least one elevated cardiovascular risk factor had significantly higher risks of death and the occurrence of a cardiovascular event, compared with women who had menopause at age 50–51 years and all risk factors at optimal levels.

In the current study, we examined the association of early menopause with risk of death and the occurrence of total CVD, fatal CVD, ischemic CVD, CHD, stroke, ischemic stroke, and hemorrhagic stroke. Zhu et al. performed a pooled analysis and found that women with premature (age < 40 years) and early menopause (age 40–44 years) had a substantially increased risk of a non-fatal CVD event before age 60 years in comparison with women who had menopause at age 50–51 years [19]. A previous review reported a modest effect of early menopause (the menopausal age categories used 50 years as a reference) on risk of CVD [7]. One meta-analysis indicated that women who experienced menopause at age < 45 years had greater risks of death and CHD than those with menopause at age ≥ 45 years; however, no significant difference in risk of CHD was found between menopause at age 45–49 years and at ≥ 50 years [6]. Another meta-analysis demonstrated a substantially increased risk of ischemic CVD following menopause at age < 40 years [20]. The results of the present study extend the previous findings and suggest that women with menopause at age 45–49 years have a higher risk of death and those with early menopause (age < 45 years) and relatively early menopause (age 45–49 years) also have a significantly higher risk of ischemic stroke than women with menopause at an average age of 50–51 years. However, this association was not found in women who experienced premature menopause (age < 40 years), probably owing to the small sample size of women with premature menopause in the current study.

The results from previous studies on the association between early menopause and stroke are inconsistent. One meta-analysis based on pooled results failed to show a significant difference in stroke risk between women with menopause at age < 45 years and those with menopause at age ≥ 45 years [6]. By contrast, the prospective longitudinal results from the Framingham Heart Study showed that menopause before age 42 years was associated with an increased risk of ischemic stroke [21]. A nationwide Swedish cohort study found a 47% increased risk of stroke among women who underwent hysterectomy plus bilateral oophorectomy before age 50 years compared with women who did not have either of these surgeries [22]. A previous review of observational studies reported that premature or early menopause was associated with an increased risk of ischemic stroke and that hormonal therapy before age 50 years may partly offset the increased risk; the authors suggested that estrogen may be a protective factor for ischemic stroke before age 50 years [23]. In the present study, we examined ischemic and hemorrhagic types of stroke, and found that menopause at age < 45 years (early menopause) or 45–49 years (relatively early menopause) was associated with elevated risk of ischemic stroke as compared with menopause at age 50–51 years. These results suggest an association between age at menopause and ischemic stroke, and have potential implications for the hypothesized association between hormonal therapy and reduced risk of ischemic stroke.

There is growing recognition of the clinical and public health importance of menopause in women. By 2030, the world population of menopausal and postmenopausal women is estimated to have increased to 1.2 billion, with 47 million women becoming menopausal each year [24]. Menopause is a sex-specific risk factor associated with reproductive aging in women [25], and early menopause is associated with future CVD events [5]. The present study identified detrimental effects on the risks of death and incident CVD events for relatively early menopause per se and combined with conventional CVD risk factors. Ovarian hormone production plays a key role in delaying the development of atherosclerosis during the reproductive years [26, 27]; subsequently, menopause induces a series of metabolic and hemodynamic changes that offset sexual hormone cardioprotective effects and accelerate the onset of cardiovascular events and peripheral vasomotor instability [28]. Although serum concentrations of TC, LDL-C, and lipoprotein rise sharply, whereas HDL-C declines gradually after menopause [29], the increased postmenopause cardiovascular morbidity and mortality cannot be fully explained by changes in plasma lipoproteins. The present findings also suggest that ovarian hormone deprivation may have a widespread effect on the cardiovascular system and a direct harmful effect on vessel-wall physiology [30].

One finding of the present study was that time since menopause was associated with risk of developing CHD but not with death and stroke after adjustment for the effects of time-varying menopause status, use of oral estrogen, BMI, smoking, SBP, DBP, FG, TC, LDL-C, HDL-C, TG, age at baseline, family history of CVD, and white blood cell count. Previous observational studies have assessed the association between time since menopause and risk of CVD outcomes, but these have shown conflicting results [31, 32]. Considering the relatively small number of studies on the association between time since menopause and CVD outcomes, as well as the possibility that previous studies have greater heterogeneity than the current study, the present findings must be interpreted with caution and confirmed in future work.

This study had several limitations. First, we did not measure individual estrogen levels and therefore did not conduct an in-depth analysis on the association of early menopause with estrogen levels, risk of death, and CVD outcomes. Second, very few women used hormone therapy at baseline, so we did not validate the timing hypothesis or window of opportunity hypothesis regarding the effects of estrogen on CVD risk by age at menopause or by age at initiation of exogenous hormone therapy. Third, smoking is a known shared risk factor for early menopause and CVD [33]. There were very low smoking rates among women in the present study, so we did not conduct separate analysis of the intermediate role of smoking on the association between age at menopause and CVD. Fourth, there was no information on premenopausal migraine, recurrent pregnancy loss and hypertensive pregnancy disorders; therefore, we did not analyze their impact on the CVD risk in the current cohort. Finally, a total of 0 (0%) and 272 (12.9%) women reported use of oral contraceptives (OCs) in the 1992 baseline survey and 2002 follow-up visit, respectively. However, there was no information on IUD use in the cohort. We found that no unanimous conclusions regarding the association between OCs and risk estimation of age at menopause could be reached. The Study of Women’s Health across the Nation (SWAN) reported that prior OC use was significantly associated with later age at natural menopause [34]. By contrast, the DOM cohort indicated that use of high-dose OCs advanced the onset of menopause by approximately 1.2 months for every year of OC use, as compared with no OC use [35]. We had zero women who used OCs at baseline according to self-reported information; therefore, we did not adjust for the use of OCs at baseline.

## Conclusions

This study demonstrated that earlier menopause had independent effects on the risk of death and ischemic stroke after adjustment for time-varying variates of menopause status, use of oral estrogen, BMI, smoking, SBP, DBP, FG, TC, LDL-C, HDL-C, and TG, as well as baseline variates of age, family history of CVD, and white blood cell count in our study population. These findings provide evidence for the effects of menopause on health and have potential implications for clinical and public health management of menopause.

## Supplementary Information

**Additional file 1: Table 1**: Hazard ratios and 95% CIs for death and cardiovascular events associated with age at menopause (sensitivity analyses). **Table 2**: Hazard ratios and 95% CIs for death and cardiovascular events associated with different menopause stages (menopausal transition) at baseline in women. **Table 3**: Hazard ratios and 95% CIs for death and cardiovascular events associated with different menopause stages (perimenopause) at baseline in women. **Figure 1**: Cumulative incidence of death and cardiovascular events according to menopause stage at baseline

## Data Availability

The datasets used and/or analyzed during the current study are available from the corresponding author on reasonable request.
