# Supplementary material for: Combined effect of menopause and cardiovascular risk factors on death and cardiovascular disease: a cohort study
Source: BMC Cardiovasc Disord. 2021 Feb 23;21:109. doi: 10.1186/s12872-021-01919-5 (PMC7903751; doi:10.1186/s12872-021-01919-5)

Supplements

| eTable 1: Hazard ratios and 95%CIs for death and cardiovascular events associated with age at menopause (sensitivity analyses) | | | | | | |
| --- | --- | --- | --- | --- | --- | --- |
| Age at menopause | Women | Events | Woman-years | Events per 1000 person-years | Adjusted HR  (95% CI) | *P*-value^a^ |
| Death |  |  |  |  |  |  |
| <45 years old | 120 | 6 | 3088 | 1.9 | 0.99 (0.40, 2.41) | 0.98 |
| 45-49 years old | 790 | 64 | 19880 | 3.2 | 2.01 (1.25, 3.23) | 0.004 |
| 50-51 years old | 506 | 25 | 13088 | 1.9 | 1.00 (reference) | <0.001 |
| >51 years old | 648 | 25 | 16767 | 1.5 | 0.79 (0.45, 1.38) | 0.41 |
| Cardiovascular disease |  |  |  |  |  |  |
| <45 years old | 120 | 16 | 2932 | 5.5 | 1.32 (0.75, 2.33) | 0.34 |
| 45-49 years old | 790 | 68 | 19200 | 3.5 | 1.18 (0.81, 1.72) | 0.38 |
| 50-51 years old | 506 | 49 | 12611 | 3.9 | 1.00 (reference) | 0.41 |
| >51 years old | 648 | 56 | 16269 | 3.4 | 0.91 (0.62, 1.34) | 0.64 |
| Fatal cardiovascular disease |  |  |  |  |  |  |
| <45 years old | 120 | 1 | 3088 | 0.3 | 0.35 (0.05, 2.75) | 0.32 |
| 45-49 years old | 790 | 14 | 19802 | 0.7 | 1.26 (0.56, 2.83) | 0.58 |
| 50-51 years old | 506 | 11 | 13038 | 0.8 | 1.00 (reference) | 0.11 |
| >51 years old | 648 | 5 | 16747 | 0.3 | 0.38 (0.13, 1.11) | 0.08 |
| Ischemic cardiovascular disease |  |  |  |  |  |  |
| <45 years old | 120 | 14 | 2956 | 4.7 | 1.35 (0.74, 2.48) | 0.33 |
| 45-49 years old | 790 | 62 | 19230 | 3.2 | 1.29 (0.86, 1.92) | 0.22 |
| 50-51 years old | 506 | 42 | 12697 | 3.3 | 1.00 (reference) | 0.39 |
| >51 years old | 648 | 51 | 16316 | 3.1 | 0.98 (0.65, 1.47) | 0.91 |
| Coronary heart disease |  |  |  |  |  |  |
| <45 years old | 120 | 7 | 3043 | 2.3 | 1.14 (0.49, 2.65) | 0.75 |
| 45-49 years old | 790 | 27 | 19692 | 1.4 | 0.82 (0.47, 1.43) | 0.48 |
| 50-51 years old | 506 | 25 | 12927 | 1.9 | 1.00 (reference) | 0.45 |
| >51 years old | 648 | 21 | 16652 | 1.3 | 0.66 (0.37, 1.18) | 0.16 |
| Stroke |  |  |  |  |  |  |
| <45 years old | 120 | 11 | 2968 | 3.7 | 1.73 (0.85, 3.51) | 0.13 |
| 45-49 years old | 790 | 43 | 19402 | 2.2 | 1.51 (0.92, 2.48) | 0.10 |
| 50-51 years old | 506 | 26 | 12804 | 2.0 | 1.00 (reference) | 0.19 |
| >51 years old | 648 | 34 | 16397 | 2.1 | 1.06 (0.63, 1.77) | 0.83 |
| Ischemic stroke |  |  |  |  |  |  |
| <45 years old | 120 | 9 | 2991 | 3.0 | 2.03 (0.91, 4.53) | 0.09 |
| 45-49 years old | 790 | 38 | 19411 | 2.0 | 2.07 (1.17, 3.68) | 0.01 |
| 50-51 years old | 506 | 18 | 12857 | 1.4 | 1.00 (reference) | 0.06 |
| >51 years old | 648 | 30 | 16431 | 1.8 | 1.36 (0.76, 2.45) | 0.30 |
| Hemorrhagic stroke |  |  |  |  |  |  |
| <45 years old | 120 | 2 | 3065 | 0.7 | 0.92 (0.20, 4.33) | 0.92 |
| 45-49 years old | 790 | 7 | 19869 | 0.4 | 0.65 (0.24, 1.81) | 0.41 |
| 50-51 years old | 506 | 9 | 13032 | 0.7 | 1.00 (reference) | 0.35 |
| >51 years old | 648 | 4 | 16733 | 0.2 | 0.34 (0.10, 1.12) | 0.08 |
| HRs: hazard ratios; CIs: confidence intervals.  Model adjusted for: time-varying covariates including menopause, use of oral estrogen due to menopause, body mass index, smoke, systolic blood pressure, diastolic blood pressure, fasting glucose, total cholesterol, low density lipoprotein cholesterol, high density lipoprotein cholesterol, and triglyceride; baseline covariates including age, family history of cardiovascular disease, and white blood cell count.  ^a^ *P* values derived from Cox regression model for non-proportional hazards. | | | | | | |

| eTable 2: Hazard ratios and 95%CIs for death and cardiovascular events associated with different menopause stage (menopausal transition) at baseline in women | | | | | | |
| --- | --- | --- | --- | --- | --- | --- |
| Menopause stages | Women | Events | Woman-years | Events per 1000 person-years | Adjusted HR  (95% CI) | *P*-value^a^ |
| Death |  |  |  |  |  |  |
| Reproductive stage | 1252 | 42 | 32366 | 1.3 | 1.00 (reference) | 0.36 |
| Menopausal transition | 238 | 10 | 6124 | 1.6 | 0.88 (0.39, 2.02) | 0.77 |
| Early postmenopause | 354 | 35 | 8859 | 4.0 | 1.56 (0.72, 3.35) | 0.26 |
| Late postmenopause | 260 | 37 | 6493 | 5.7 | 1.81 (0.69, 4.75) | 0.23 |
| Cardiovascular disease |  |  |  |  |  |  |
| Reproductive stage | 1252 | 70 | 31777 | 2.2 | 1.00 (reference) | 0.10 |
| Menopausal transition | 238 | 22 | 5887 | 3.7 | 1.18 (0.66, 2.12) | 0.58 |
| Early postmenopause | 354 | 48 | 8429 | 5.7 | 1.59 (0.88, 2.88) | 0.12 |
| Late postmenopause | 260 | 56 | 5881 | 9.5 | 2.32 (1.12, 4.80) | 0.02 |
| Fatal cardiovascular disease |  |  |  |  |  |  |
| Reproductive stage | 1252 | 6 | 32326 | 0.2 | 1.00 (reference) | 0.48 |
| Menopausal transition | 238 | 2 | 6124 | 0.3 | 1.07 (0.16, 7.28) | 0.94 |
| Early postmenopause | 354 | 9 | 8819 | 1.0 | 2.31 (0.41, 12.92) | 0.34 |
| Late postmenopause | 260 | 16 | 6418 | 2.5 | 3.72 (0.48, 28.75) | 0.21 |
| Ischemic cardiovascular disease |  |  |  |  |  |  |
| Reproductive stage | 1252 | 61 | 31890 | 1.9 | 1.00 (reference) | 0.09 |
| Menopausal transition | 238 | 21 | 5911 | 3.6 | 1.30 (0.71, 2.37) | 0.40 |
| Early postmenopause | 354 | 43 | 8439 | 5.1 | 1.68 (0.90, 3.12) | 0.10 |
| Late postmenopause | 260 | 51 | 5920 | 8.6 | 2.52 (1.18, 5.40) | 0.02 |
| Coronary heart disease |  |  |  |  |  |  |
| Reproductive stage | 1252 | 31 | 32186 | 1.0 | 1.00 (reference) | 0.02 |
| Menopausal transition | 238 | 5 | 6104 | 0.8 | 0.74 (0.26, 2.14) | 0.58 |
| Early postmenopause | 354 | 28 | 8661 | 3.2 | 2.87 (1.24, 6.65) | 0.01 |
| Late postmenopause | 260 | 20 | 6372 | 3.1 | 2.96 (0.99, 8.85) | 0.05 |
| Stroke |  |  |  |  |  |  |
| Reproductive stage | 1252 | 39 | 31999 | 1.2 | 1.00 (reference) | 0.08 |
| Menopausal transition | 238 | 17 | 5907 | 2.9 | 1.67 (0.81, 3.48) | 0.17 |
| Early postmenopause | 354 | 22 | 8619 | 2.6 | 1.29 (0.57, 2.93) | 0.54 |
| Late postmenopause | 260 | 39 | 6017 | 6.5 | 2.67 (1.02, 6.96) | 0.04 |
| Ischemic stroke |  |  |  |  |  |  |
| Reproductive stage | 1252 | 31 | 32069 | 1.0 | 1.00 (reference) | 0.05 |
| Menopausal transition | 238 | 16 | 5931 | 2.7 | 1.84 (0.84, 4.00) | 0.13 |
| Early postmenopause | 354 | 17 | 8630 | 2.0 | 1.14 (0.46, 2.81) | 0.78 |
| Late postmenopause | 260 | 34 | 6031 | 5.6 | 2.65 (0.94, 7.49) | 0.07 |
| Hemorrhagic stroke |  |  |  |  |  |  |
| Reproductive stage | 1252 | 8 | 32295 | 0.2 | 1.00 (reference) | 0.70 |
| Menopausal transition | 238 | 2 | 6098 | 0.3 | 1.49 (0.22, 10.11) | 0.69 |
| Early postmenopause | 354 | 5 | 8849 | 0.6 | 2.52 (0.38, 16.77) | 0.34 |
| Late postmenopause | 260 | 7 | 6476 | 1.1 | 3.73 (0.37, 37.17) | 0.26 |
| HRs: hazard ratios; CIs: confidence intervals.  Model adjusted for: time-varying covariates including menopause, use of oral estrogen due to menopause, body mass index, smoke, systolic blood pressure, diastolic blood pressure, fasting glucose, total cholesterol, low density lipoprotein cholesterol, high density lipoprotein cholesterol, and triglyceride; baseline covariates including age, family history of cardiovascular disease, and white blood cell count.  ^a^ *P* values derived from Cox regression model for non-proportional hazards. | | | | | | |

| eTable 3: Hazard ratios and 95%CIs for death and cardiovascular events associated with different menopause stage (perimenopause) at baseline in women | | | | | | |
| --- | --- | --- | --- | --- | --- | --- |
| Menopause stages | Women | Events | Woman-years | Events per 1000 person-years | Adjusted HR  (95% CI) | *P*-value^a^ |
| Death |  |  |  |  |  |  |
| Reproductive stage | 1252 | 42 | 32366 | 1.3 | 1.00 (reference) | 0.60 |
| Perimenopause | 309 | 17 | 7879 | 2.2 | 1.07 (0.51, 2.23) | 0.86 |
| Early postmenopause | 283 | 28 | 7104 | 3.9 | 1.47 (0.66, 3.30) | 0.35 |
| Late postmenopause | 260 | 37 | 6493 | 5.7 | 1.73 (0.65, 4.55) | 0.27 |
| Cardiovascular disease |  |  |  |  |  |  |
| Reproductive stage | 1252 | 70 | 31777 | 2.2 | 1.00 (reference) | 0.15 |
| Perimenopause | 309 | 32 | 7567 | 4.2 | 1.30 (0.75, 2.23) | 0.35 |
| Early postmenopause | 283 | 38 | 6749 | 5.6 | 1.49 (0.80, 2.79) | 0.21 |
| Late postmenopause | 260 | 56 | 5881 | 9.5 | 2.22 (1.07, 4.60) | 0.03 |
| Fatal cardiovascular disease |  |  |  |  |  |  |
| Reproductive stage | 1252 | 6 | 32326 | 0.2 | 1.00 (reference) | 0.63 |
| Perimenopause | 309 | 4 | 7876 | 0.5 | 1.50 (0.28, 7.89) | 0.64 |
| Early postmenopause | 283 | 7 | 7067 | 1.0 | 1.95 (0.33, 11.63) | 0.46 |
| Late postmenopause | 260 | 16 | 6418 | 2.5 | 3.28 (0.42, 25.53) | 0.26 |
| Ischemic cardiovascular disease |  |  |  |  |  |  |
| Reproductive stage | 1252 | 61 | 31890 | 1.9 | 1.00 (reference) | 0.11 |
| Perimenopause | 309 | 29 | 7593 | 3.8 | 1.38 (0.78, 2.44) | 0.27 |
| Early postmenopause | 283 | 35 | 6756 | 5.2 | 1.64 (0.86, 3.15) | 0.14 |
| Late postmenopause | 260 | 51 | 5920 | 8.6 | 2.48 (1.15, 5.32) | 0.02 |
| Coronary heart disease |  |  |  |  |  |  |
| Reproductive stage | 1252 | 31 | 32186 | 1.0 | 1.00 (reference) | 0.05 |
| Perimenopause | 309 | 10 | 7846 | 1.3 | 1.14 (0.48, 2.70) | 0.77 |
| Early postmenopause | 283 | 23 | 6919 | 3.3 | 2.83 (1.18, 6.76) | 0.02 |
| Late postmenopause | 260 | 20 | 6372 | 3.1 | 2.84 (0.95, 8.43) | 0.06 |
| Stroke |  |  |  |  |  |  |
| Reproductive stage | 1252 | 39 | 31999 | 1.2 | 1.00 (reference) | 0.05 |
| Perimenopause | 309 | 23 | 7592 | 3.0 | 1.69 (0.84, 3.41) | 0.14 |
| Early postmenopause | 283 | 16 | 6934 | 2.3 | 1.07 (0.45, 2.58) | 0.88 |
| Late postmenopause | 260 | 39 | 6017 | 6.5 | 2.46 (0.94, 6.47) | 0.07 |
| Ischemic stroke |  |  |  |  |  |  |
| Reproductive stage | 1252 | 31 | 32069 | 1.0 | 1.00 (reference) | 0.05 |
| Perimenopause | 309 | 20 | 7619 | 2.6 | 1.75 (0.82, 3.75) | 0.19 |
| Early postmenopause | 283 | 13 | 6941 | 1.9 | 1.03 (0.39, 2.70) | 0.95 |
| Late postmenopause | 260 | 34 | 6031 | 5.6 | 2.58 (0.91, 7.34) | 0.08 |
| Hemorrhagic stroke |  |  |  |  |  |  |
| Reproductive stage | 1252 | 8 | 32295 | 0.2 | 1.00 (reference) | 0.76 |
| Perimenopause | 309 | 4 | 7850 | 0.5 | 2.04 (0.38, 10.88) | 0.41 |
| Early postmenopause | 283 | 3 | 7097 | 0.4 | 1.46 (0.19, 11.53) | 0.72 |
| Late postmenopause | 260 | 7 | 6476 | 1.1 | 2.78 (0.28, 28.02) | 0.39 |
| HRs: hazard ratios; CIs: confidence intervals.  Model adjusted for: time-varying covariates including menopause, use of oral estrogen due to menopause, body mass index, smoke, systolic blood pressure, diastolic blood pressure, fasting glucose, total cholesterol, low density lipoprotein cholesterol, high density lipoprotein cholesterol, and triglyceride; baseline covariates including age, family history of cardiovascular disease, and white blood cell count.  ^a^ *P* values derived from Cox regression model for non-proportional hazards. | | | | | | |


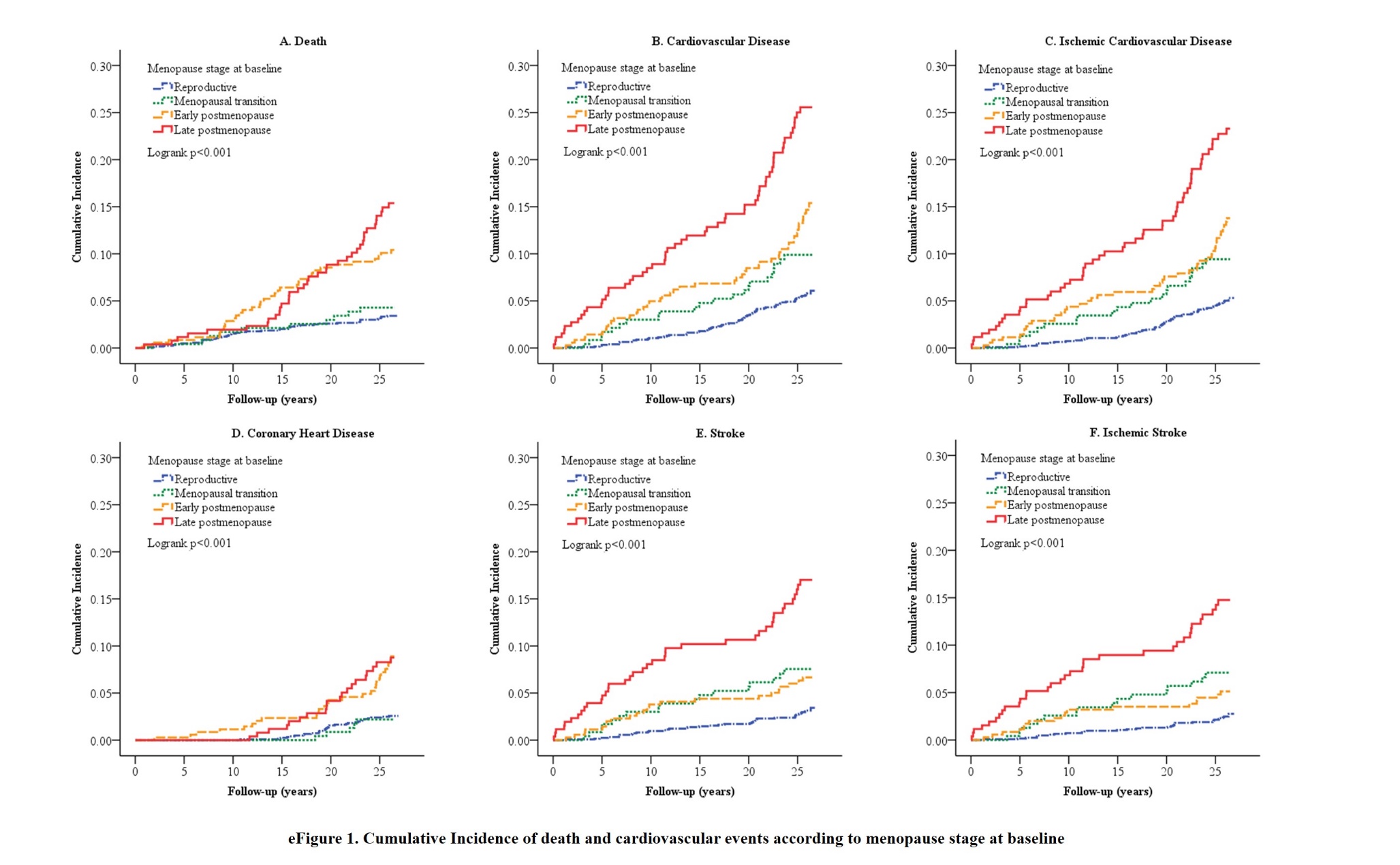

Supplement: Supplementary file 1 — Additional file 1: Table 1: Hazard ratios and 95% CIs for death and cardiovascular events associated with age at menopause (sensitivity analyses). Table 2: Hazard ratios and 95% CIs for death and cardiovascular events associated with different menopause stages (menopausal transition) at baseline in women. Table 3: Hazard ratios and 95% CIs for death and cardiovascular events associated with different menopause stages (perimenopause) at baseline in women. Figure 1: Cumulative incidence of death and cardiovascular events according to menopause stage at baseline [file 12872_2021_1919_MOESM1_ESM.docx]
